# The Association Between Cervical Human Papillomavirus Infection and Subsequent HIV Acquisition in Tanzanian and Ugandan Women: A Nested Case-Control Study

**DOI:** 10.1093/infdis/jiw094

**Published:** 2016-03-06

**Authors:** Katherine E. Gallagher, Kathy Baisley, Heiner Grosskurth, Andrew Vallely, Saidi Kapiga, Judith Vandepitte, Anatoli Kamali, Silvia De Sanjosé, John Changalucha, Richard Hayes, Deborah Watson-Jones

**Affiliations:** 1 Clinical Research Department, Faculty of Infectious and Tropical Diseases; 2 Department of Infectious Disease Epidemiology, Faculty of Epidemiology and Population Health, London School of Hygiene and Tropical Medicine, United Kingdom; 3 Mwanza Intervention Trials Unit; 4 Mwanza Research Center, National Institute for Medical Research, Tanzania; 5 Public Health Interventions Research Group, Kirby Institute, University of New South Wales, Sydney, Australia; 6 MRC/UVRI Uganda Research Unit on AIDS, Entebbe, Uganda; 7 Cancer Epidemiology Research Program, Catalan Institute of Oncology, IDIBELL, CIBERESP, Barcelona, Spain

**Keywords:** human papillomavirus, HIV (transmission, prevention), Sub-Saharan Africa

## Abstract

***Objective*** This study was performed to analyze the associations between cervical human papillomavirus (HPV) infection and human immunodeficiency virus (HIV) acquisition, using cervical samples from previous studies in Tanzania and Uganda.

***Methods.*** A total of 161 adult women who acquired HIV infection during follow-up and 464 individually matched HIV-seronegative controls were selected from 5 cohorts of women working in bars and recreational facilities. Stored cervical samples were tested for 37 HPV genotypes, using a polymerase chain reaction assay (Roche Linear Array genotyping assay). Multivariate matched analysis using conditional logistic regression was performed to evaluate HPV infection, persistence, and clearance as predictors of HIV acquisition.

***Results.*** HIV seroconverters were significantly more likely than controls to frequently drink alcohol and to be infected with *Chlamydia trachomatis, Neisseria* g*onorrhoeae*, or herpes simplex virus type 2. There was no evidence of an association between HIV acquisition and any detectable HPV at the visit prior to HIV seroconversion (adjusted odds ratio, 1.02; 95% confidence interval, .66–1.57) or between HIV acquisition and persistent HPV infection (defined as 2 positive HPV genotype–specific test results at least 6 months apart), cleared HPV infection (defined as a positive HPV test result followed by negative HPV genotype–specific test result), or newly acquired HPV infection, compared with HPV-negative women.

***Conclusions.*** There was no evidence of association between HPV infection status and subsequent HIV acquisition. These results stand in contrast to other observational studies.

Human papillomavirus (HPV) is a highly prevalent sexually transmitted virus. There are 40 genotypes that infect the genital mucosa [1], 13 of which are regarded as carcinogenic [2]. Persistent infection with carcinogenic HPV (ie, high-risk HPV [HR-HPV]) genotypes is the cause of almost all cases of cervical cancer [3], the third most common cancer among women worldwide [4]. HPV DNA has also been associated with cancerous lesions of the vagina, vulva, penis, anus, and oropharynx [3, 5]. Bivalent (Cervarix; covering HPV 16 and 18) and quadrivalent (Gardasil; covering HPV 6, 11, 16, and 18) HPV vaccines are available. A nonavalent vaccine (covering HPV 6, 11, 16, 18, 31, 33, 45, 52, and 58) gained Food and Drug Administration approval in December 2014 [6]. More than 80% of men and women contract HPV at least once in their lifetime [3, 7]. However, only a small proportion of these infections persist to cause lesions, which can progress to cancer [8, 9], and 90% of infections are cleared within 2 years [10].

East African women harbor the highest HPV DNA prevalence in the world (31.7%; 95% confidence interval [CI], 29.5–33.8) and experience the highest age-standardized incidence rate of cervical cancer (42.7 cases/100 000 women/year) [4, 11–13]. Among cytologically normal, human immunodeficiency virus (HIV)–negative, young women with a median age of 18 years, 73.5% in Tanzania [14] and 73.2% in Uganda [15] were found to have detectable HPV DNA of any genotype. Detection of HPV DNA increases rapidly after HIV seroconversion in HIV-infected women, compared with detection rates over time in women who remain HIV uninfected [16, 17]. HPV is more likely to persist and to progress to cancer in HIV-positive and immunosuppressed women [18, 19].

There is growing evidence that HPV may be an important cofactor in HIV acquisition. There is a plausible biological mechanism for this association: weakened cell adhesion caused by HPV infection could expose basal layers of the epithelium in the genital tract and form additional HIV entry points [20, 21]. HPV infection and the mechanisms involved in its clearance may cause an influx of inflammatory cytokines [21], macrophages, and T cells [22], creating a favorable environment for HIV invasion. A meta-analysis found an association between detectable HPV DNA and HIV acquisition in 7 of 8 observational studies of both men and women [23, 24–29]; there was a 2-fold increased risk of HIV acquisition among women with prevalent HPV infection with any HPV genotype (hazard ratio, 2.06; 95% CI, 1.44–2.94). Similar associations were observed for infection with HR HPV and low-risk HPV (LR-HPV) genotypes and in 2 studies analyzing the clearance of any genotype [27, 28], but no association was found with HPV persistence. Since this meta-analysis, 2 further observational studies in South Africa that investigated cervical samples confirmed the association between any HPV infection and HIV acquisition in women [30, 31]. Two studies involving men have shown a positive association between penile HPV clearance and HIV acquisition [32, 33]. Clearance of penile HPV infections was associated with elevated dendritic cell density in the foreskin epidermis [33].

In this article, we present findings from a nested case-control study undertaken to investigate the association between cervical HPV infection and HIV acquisition, using stored cervical samples from 5 previous studies in the Lake Zone and Kilimanjaro regions of Tanzania (4 studies) and Kampala, Uganda (1 study). The extent to which HPV infection, persistence, and clearance appear to be predictors for HIV infection is described.

## METHODS

### Study Participants and Design

Cases (women who acquired HIV infection after enrollment) and controls (women who remained HIV negative) were selected from 5 previously enrolled cohorts of women aged 16–45 years working in bars, guesthouses, and recreational facilities in urban and semiurban areas of Tanzania and Uganda (Table 1). Recruitment methods for these studies have been described previously [34–37]. All studies recruited women from their place of work, followed them for ≥12 months between 2002 and 2011, and measured the incidence of HIV infection and other sexually transmitted infections (STIs) at 3–6-month intervals. There was good retention of participants at 12 months across all studies (range, 68% [36] to 90% [37]). Participants were interviewed at enrollment and follow-up visits on socioeconomic status, education level, alcohol and drug usage, sexual behavior, and family-planning practices.

**Table 1.** Summary of 5 Cohort Studies From East Africa That Were Used for the Nested Case-Control Study of the Association Between Human Papillomavirus Infection and Human Immunodeficiency Virus (HIV) Acquisition

Study (Location)	Population	HIV-Negative Enrollees, No.	Period	Follow-up Duration, mo	Relevant Sample Types	Sample Collection Frequency	Cases (n = 161)	Controls (n = 464)
HSV2 Suppressive Treatment Trial (Lake Zone, Tanzania)	Women working in settings such as bars and guesthouses; age 16–35 y	821	2003–2007	30	Blood, vaginal swab, cervical swab, CVL	Blood: every 3 mo; cervical-vaginal: 0, 6, 12, 24, 30 mo	54	154
Microbicide Feasibility Cohort (Lake Zone)	Women working in settings such as bars and guesthouses; age 16–54 y	1156	2002–2004	12	Blood, vaginal swab, cervical swab	0, 6, 12 mo (and at 3 and 9 mo for those with symptoms of HIV infection/STIs)	19	56
Women's Health Project (Lake Zone)	Women working in settings such as bars and guesthouses; age 18–44 y	966	2008–2009	12	Blood, vaginal swab, cervical swab, CVL	Blood: 0, 3, 6, 9, 12; cervical-vaginal: 0, 6, 12 mo	28	82
EDCTP Vaccine Cohort (Kilimanjaro Region, Tanzania)	Women working in settings such as bars and guesthouses; age 18–44 y	412	2009–2011	12	Blood, vaginal swab, cervical swab	0, 3, 6, 9, 12 mo	14	39
Good Health for Women, (Kampala, Uganda)	Self-reported female sex workers and women working in entertainment; age ≥18 y	646	2008–2011	18	Blood, vaginal swab, cervical swab	0, 3, 6, 9, 12, 18 mo (blood was also collected at 15 mo)	46	133

Abbreviations: CVL, cervico-vaginal lavage; STI, sexually transmitted infection.

Across the 5 studies, 178 women were HIV negative at enrollment and seroconverted to HIV during follow-up; of these, 172 attended at least 1 study visit ≤12 months before the first detection of HIV and were selected as cases. Cases were individually matched with 3 randomly selected controls from the same study who attended the same follow-up visits but remained HIV seronegative at the time points selected and at the first visit after the visits selected for analysis. Controls could become HIV seropositive at a later visit. Of the 688 women from the original sample selection list, 664 (166 cases and 498 controls [96.5%]) contributed stored samples for testing; samples from 6 cases could not be located in the laboratory archives. If control samples were missing, substitute controls were selected. Some HPV test results were invalid; therefore, the final sample size comprised 161 cases and 464 controls.

Ethics approval for the 4 studies in Tanzania had been granted by the Medical Research Coordinating Committee of Tanzania. The Ugandan study had received approval from the Science and Ethics Committee of the Ugandan Virus Research Institute and the Uganda National Committee for Science and Technology. All 5 studies were approved by the ethics committee of the London School of Hygiene and Tropical Medicine. All participants provided consent for the storage and potential further analysis of their information and samples.

### Laboratory Methods

None of the studies had previously tested samples for HPV. Cervical samples from study visits before HIV seroconversion and when HIV was first detected, as well as those from the equivalent visits in controls, were tested for detectable HPV DNA, using the Linear Array HPV genotyping assay (Roche, Pleasanton, California) [38]. This test detects 13 HR-HPV types (16, 18, 31, 33, 35, 39, 45, 51, 52, 56, 58, 59, and 68) and 24 LR-HPV types (6, 11, 26, 40, 42, 53, 54, 55 [also known as 44], 61, 62, 64 [also known as 34], 66, 67, 69, 70, 71, 72, 73, 81, 82, IS39, 83, 84, and CP6108 [also known as 89]) [4]. As IS39 is regarded as a subtype of HPV 82, these genotype-specific results were combined during analysis into just 1 variable for HPV 82 [1]. Cervical samples were either extracts of buffer in which cervical swabs were washed (4 studies) or a supernatant aliquot from a cervico-vaginal lavage (CVL) specimen (1 study), depending on the availability of stored samples. HPV genotyping was performed in the laboratory of the National Institute for Medical Research (NIMR), Mwanza, according to the manufacturer's instructions. External quality assurance was performed by the Catalan Institute of Oncology (ICO), Barcelona, Spain. A random selection of 102 samples (5%) was tested by both laboratories. There was good agreement in the detection of any HPV (κ = 0.69; Supplementary Table 1).

Data on HIV infection and STIs were obtained from the original study databases. All studies used >1 test for confirmation of HIV results. The HSV-2 Suppressive Treatment Trial performed parallel enzyme-linked immunosorbent assays (ELISAs; Vironosticka Uni-Form II Ag/Ab [bioMerieux, Marcy l'Etoile, France] and Murex HIV Ag/Ab Combination [Murex Biotech, Dartford, UK]). If results were discordant or indeterminate, HIV-1 p24 antigen enzyme immunoassay (EIA; Biorad Genetic Systems, Hemel Hempstead, UK) was used to detect acute infection. A negative or indeterminate result with the p24 antigen EIA was confirmed by Western blot [34]. The Microbicide Feasibility Study used sequential testing on a gelatin-particle agglutination test (Serodia HIV-1/2; Fujirebio, Tokyo, Japan), followed by a confirmatory ELISA (Vironosticka Uni-Form II) [36]. For both the Mwanza Women's Health cohort and the Moshi Vaccines cohort, parallel testing with rapid diagnostic tests (RDTs) was performed (SD Bioline HIV-1/2 3.0 [Standard Diagnostics, Gyeonggi-do, Republic of Korea] and Determine HIV-1/2 [Alere Medical, Waltham, Massachusetts]); discordant results were confirmed by ELISA (Vironosticka Uniform II plus O and Murex HIV 1.2); discordant EIA results were confirmed by Western blot (INNO-LIA; Innogenetics, Ghent, Belgium) [37]. The Uganda Good Health for Women project tested women using a single Determine rapid diagnostic test (Abbott Determine HIV-1/2) with confirmation of positive results using parallel ELISAs (Vironosticka Uni-Form II plus O, Murex HIV 1.2.O). Discordant results were confirmed by Western blot (Cambridge Calypte, Portland, Oregon) [35].

Serological tests for syphilis were analyzed by a rapid plasma regain (RPR) test (Biotec, Birdport, UK) and the *Treponema pallidum* hemagglutination (TPHA) test (Biotec). Results from both tests were used to define active syphilis (positive results of both tests), past or treated syphilis (a negative result of the RPR test and a positive result of the TPHA test), and negative for syphilis (negative results of both tests or a positive result of the RPR test and a negative result of the TPHA test) in all but 1 study. One study only performed the TPHA test if participants had a positive result of the RPR test. All studies tested serum samples for antibodies to herpes simplex virus type 2 (HSV-2), using a type-specific immunoglobulin ELISA (Kalon Biologicals, Guildford, UK).

Cervical swabs were tested by polymerase chain reaction analysis for *Neisseria gonorrhoeae* and *Chlamydia trachomatis* (Amplicor; Roche). Vaginal swabs were used to culture *Trichomonas vaginalis*, using In-Pouch (Biomed Diagnostics, White City, Oregon), and to prepare a Gram-stained slide for the detection of bacterial vaginosis, using the Nugent score.

### Statistical Methods

**Figure 1.**
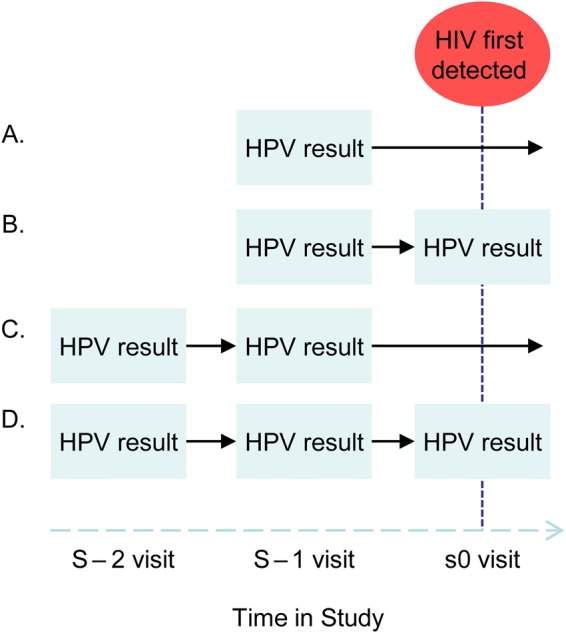
Combinations of original study visits and available human papillomavirus (HPV) test results, relative to the visit at which human immunodeficiency virus (HIV) was first detected, attended by cases and controls and selected for analysis. The s − 1 visit denotes the visit preceding the visit during which HIV seroconversion was detected, and the s − 2 visit denotes the visit preceding the s − 1 visit.

Conditional logistic regression was used to analyze associations between HIV acquisition and (1) the presence of an HPV genotype–specific infection at the visit prior to the HIV seroconversion visit (the s − 1 visit), or at the equivalent visit in controls, (2) evidence of any HPV clearance, persistent HPV infection only, or HPV acquisition only. Clearance was defined as a genotype-specific positive test result followed by a negative test result for the same genotype, regardless of the status of other HPV coinfections. Persistence was defined as detectable infection with the same genotype for ≥6 months with no evidence of clearance of other genotypes. Acquisition was defined as a negative HPV test result followed by a positive HPV test result for the same genotype and no evidence of clearance or persistence of other genotypes. The possible combinations of visits attended by the cases and controls selected for this study and visit classifications are detailed in Figure 1. Owing to variations in questionnaires, a number of variables identified a priori as potential confounders were recoded to be consistent across studies (Table 2).

**Table 2.** Characteristics of the Matched Cases and Controls and Age-Adjusted Associations With Case-Control Status, Using Conditional Logistic Regression

Characteristic	Cases, No. (%) (n = 161)	Controls, No. (%) (n = 464)	Age-Adjusted OR (95% CI)	*P* Value
Enrollment data				
Age group, y				
16–24	79 (49.0)	178 (38.4)	1	<.001^a^
25–34	70 (43.5)	208 (44.8)	0.64 (.41–.99)	
≥35	12 (7.5)	78 (16.8)	0.28 (.13–.57)	
Marital status				
Married/living as married	27 (16.8)	109 (23.5)	1	.221
Separated/divorced/widowed	94 (58.4)	252 (54.3)	1.54 (.92–2.57)	
Single	40 (24.8)	103 (22.2)	1.22 (.66–2.25)	
Religion				
Christian	121 (75.2)	326 (70.3)	1	.495
Muslim	34 (21.1)	121 (26.1)	0.78 (.49–1.23)	
Other/no religion	6 (3.7)	17 (3.6)	1.21 (.46–3.19)	
Main occupation				
Restaurant/bar worker/cleaner	85 (52.8)	247 (53.2)	1	.916
Local brew or street food vendor	40 (24.8)	113 (24.4)	1.05 (.64–1.74)	
Manager/owner/office worker	8 (5.0)	34 (7.3)	0.79 (.33–1.89)	
Sex worker^b^	18 (11.2)	46 (9.9)	1.22 (.59–2.53)	
None/other	10 (6.2)	24 (5.2)	1.26 (.56–2.80)	
Maximum education level				
None/incomplete primary	48 (29.8)	162 (34.9)	1	.008
Complete primary	92 (57.1)	214 (46.1)	1.61 (1.04–2.51)	
Some secondary/higher	21 (13.0)	88 (19.0)	0.66 (.36–1.23)	
Crowding^c^				
0–2	95 (56.9)	248 (50.5)	1	.116^a^
3–4	45 (27.0)	129 (26.3)	0.64 (.41–.99)	
≥5	27 (16.2)	114 (23.2)	0.29 (.14–.59)	
Lifetime no. of sex partners^d^				
<5	50 (31.9)	179 (39.5)	1	.024^a^
5–9	39 (24.8)	92 (20.3)	1.71 (1.02–2.87)	
≥10	24 (15.3)	80 (17.7)	1.49 (.81–2.75)	
Do not remember	44 (28.1)	102 (22.5)	2.78 (1.33–5.82)	
Forced sex^e^				
No	121 (77.1)	378 (83.1)	1	.058
Yes	36 (22.9)	77 (16.9)	1.79 (.98–3.23)	
Alcohol consumption				
Never	53 (32.9)	193 (41.6)	1	.003^a^
1 time/wk or less	9 (5.6)	52 (11.2)	0.60 (.28–1.32)	
2–3 times/wk	47 (29.2)	114 (24.6)	1.75 (1.06–2.91)	
≥4 times/wk	52 (32.3)	105 (22.6)	2.02 (1.23–3.33)	
Time-updated data				
Current method of family planning^f^				
Nothing	50 (31.1)	143 (31.4)	1	.650
Oral pill	29 (18.0)	84 (18.5)	1.04 (.58–1.86)	
Injection	45 (28.0)	100 (22.0)	1.30 (.77–2.17)	
Condom	30 (18.6)	103 (22.6)	0.83 (.48–1.43)	
Other (eg, traditional/calendar)	7 (4.4)	25 (5.5)	0.93 (.34–2.57)	
Condom use at last sex^g^				
No	87 (55.4)	268 (60.1)	1	.439
Yes	70 (44.6)	178 (39.9)	1.16 (.79–1.71)	
Transactional sex^h^				
No	85 (53.1)	290 (63.7)	1	.002
Yes	75 (46.9)	165 (36.3)	2.18 (.37–.98)	
Partners in the last 3 mo				
None/1	94 (58.8)	298 (65.5)	1	.148^a^
2–9	39 (24.4)	89 (19.6)	1.50 (.91–2.47)	
≥10	18 (11.3)	54 (11.9)	1.30 (.58–2.93)	
Do not remember	9 (5.6)	14 (3.1)	2.52 (.90–7.00)	
STI test results at the s0 and s − 1 visits				
*C. trachomatis*				
Negative at both visits	133 (82.6)	431 (92.9)	1	<.001
Positive at ≥1 visit	28 (17.4)	33 (7.1)	2.76 (1.57–4.87)	
*N. gonorrhoeae*				
Negative at both visits	130 (80.8)	426 (91.8)	1	<.001
Positive at ≥1 visit	31 (19.3)	38 (8.2)	2.58 (1.51–4.41)	
HSV-2^i^				
Negative at both visits	15 (9.4)	70 (15.2)	1	.022
Positive at ≥1 visit	144 (90.6)	392 (84.9)	2.00 (1.08–3.75)	

Abbreviations: CI, confidence interval; *C. trachomatis*, *Chlamydia trachomatis*; HSV-2, herpes simplex virus type 2; *N. gonorrhoeae, Neisseria gonorrhoeae*; OR, odds ratio; s0 visit, the visit during which HIV seroconversion was detected; s − 1 visit, the visit preceding the visit during which HIV seroconversion was detected STI, sexually transmitted infection.

^a^ For linear trend. In the association with number of partners, we assume that women who stated “do not remember” had the highest number of partners.

^b^ Only the Ugandan study included sex work as a category in answer to the question “What is your main source of income?”

^c^ Data are no. of people living in the participant's residence^.^

^d^ Data for 15 women (11 [2.4%] of controls and 4 [2.5%] of cases) were missing.

^e^ Data are for experience of forced sex during the 3 mo before enrollment. Data for 13 women (9 [1.9%] of controls and 4 [2.5%] of cases) were missing.

^f^ Data for 9 women (1.9% controls) were missing.

^g^ Data for 22 women (18 [3.8%] of controls and 4 [2.5%] of cases) were missing.

^h^ Data are for experience of transactional sex during the 3 mo before enrollment. Data for 10 women (1 case [<1%] and 9 controls [2%]) were missing.

^i^ Data for 4 women (2 cases [1%] and 2 controls [<1%]) were missing.

First, age-adjusted analyses, with age as a categorical variable, were performed on all available covariates identified a priori as potential confounders and common to all study questionnaires, to assess their association with HIV acquisition (Table 2). Information on sociodemographic and behavioral covariates was either from baseline (the enrollment questionnaire) or time-updated to values at the time of first detection of HIV seroconversion (the index visit; s0). Since STI data were not gathered at every visit, time-updated STI results used data from both the s0 visit and the s − 1 visit; women were classified as positive for the STI if they had a positive test result at either time point.

Next, age-adjusted analyses of the association of HPV with HIV acquisition were performed. Multivariable models to control for potential confounding were then constructed. Variables describing mobility, vaginal cleansing practices, *Trichomonas* infection, and presence of genital warts were not included in the multivariable analysis because of missing data. The remaining covariates were assessed iteratively for evidence of a change in the effect estimate for the association of HPV and HIV, starting with covariates with the strongest association with HIV in age-adjusted analyses. To minimize the number of parameters estimated in the model, only covariates that influenced the size of the effect estimate for HPV remained in the multivariate model. Multicollinearity was assessed against a model adjusted only for age. All *P* values were generated by likelihood ratio tests. Assuming an HPV prevalence of 30%–50% in control subjects, 150 HIV-positive cases and 450 HIV-negative controls, we calculated that we would have ≥80% power to detect an odds ratio (OR) of ≥1.75 for the association between HIV acquisition and HPV infection at the visit before HIV seroconversion.

## RESULTS

Across all time points, 90 of the 1258 samples (7%) returned an invalid/inhibited HPV result on first testing. Sixty-six of these samples had sufficient volume to be retested; 41 gave a valid HPV result. Valid HPV results were therefore available for 1209 samples (96%). At the s − 1 time point, 625 of 664 women (94%; 161 cases and 464 controls) returned valid HPV test results and therefore contributed to the main analysis of the association between HIV status and HPV prevalence.

The mean age of cases and controls at enrollment was 25 years (range 16–45 years; Table 2). One fifth of women (22%) were married or living as married. Over 50% of women stated that their main source of income came from restaurant or bar or guesthouse work and 24% identified themselves as local brew or street food sellers. A proportion of those whose income source was categorized as “other” may have been sex workers, as this was only determined as a separate category in the Ugandan study. Although 49% had completed primary education, <18% had some secondary or higher education. In time-updated data from the visit of first detection of HIV (s0) approximately one third of women reported not currently using any form of contraception, whereas 57% reported having used a condom at last sex.

Younger age was strongly associated with HIV seroconversion (*P*_trend_ < .001; Table 2). In age-adjusted analysis, women reporting a higher number of lifetime partners, having been paid for sex in the past 3 months, and those drinking alcohol more frequently, had a significantly increased odds of HIV seroconversion. Women who had completed primary education had a higher risk than those with incomplete primary education. Women who experienced forced sex during the 3 months prior to enrollment had a higher risk of HIV seroconversion, but this effect was not statistically significant. At the s0 or the s − 1 visit, 10% of cases and controls had *C. trachomatis* infection, 11% had *N. gonorrhoeae* infection, and 86% had evidence of exposure to HSV-2. These infections significantly increased the odds of HIV seroconversion by 2.8-, 2.6-, and 2.0-fold, respectively (Table 2).

### HPV Prevalence Before HIV Acquisition

The median interval between the s − 1 visit and the first visit at which HIV was detected (the s0 visit) was 3 months; 87% of cases and controls had a cervical sample at or <6 months prior to the s0 visit.

At the s − 1 visit, 49% of women (51.6% of cases and 48.5% of controls) across groups A–D in Figure 1 had detectable HPV infection of any genotype. In adjusted analyses, there was no evidence that prevalent infection with any HPV genotype at the visit prior to the first detection of HIV was associated with HIV acquisition (adjusted OR for any HPV type infection, 1.02; 95% CI, .66–1.57; Table 3).

**Table 3.** Associations Between Human Papillomavirus (HPV) Infection at the Visit Preceding the First Detection of Human Immunodeficiency Virus (HIV) Seroconversion (the s − 1 Visit) and Subsequent HIV Acquisition Among Women With a Valid HPV Test Result at the s − 1 Visit

HPV Infection Status at s − 1	Cases, No. (%) (n = 161)	Controls, No. (%) (n = 464)	Age-Adjusted Analysis		Adjusted Analysis 1^a^		Adjusted Analysis 2^b^	
			OR (95% CI)	*P* Value	OR (95% CI)	*P* Value	OR (95% CI)	*P* Value
Any type								
No infection	78 (48.5)	239 (51.5)	1	.783	1	.923	1	.926
Any HPV infection	83 (51.6)	225 (48.5)	1.06 (.72–1.56)		1.02 (.68–1.53)		1.02 (.66–1.57)	
Nonavalent vaccine types								
No HPV infection	78 (48.5)	239 (51.5)	1	.867	1	.875	1	.901
Nonavalent vaccine–type HPV infection	38 (23.6)	103 (22.2)	0.99 (.61–1.60)		0.95 (.57–1.56)		0.95 (.56–1.61)	
Other HPV infection	45 (28.0)	122 (26.3)	1.12 (.71–1.75)		1.09 (.68–1.75)		1.08 (.66–1.78)	
HR/LR-HPV infection								
No HPV infection	78 (48.5)	239 (51.5)	1	.667	1	.738	1	.658
HR-HPV infection only	27 (16.8)	70 (15.1)	1.08 (.63–1.85)		1.07 (.60–1.89)		1.15 (.63–2.08)	
LR-HPV infection only	33 (20.5)	83 (17.9)	1.26 (.75–2.12)		1.19 (.69–2.06)		1.17 (.66–2.08)	
HR/LR-HPV coinfection	23 (14.3)	72 (15.5)	0.85 (.48–1.48)		0.82 (.45–1.47)		0.78 (.43–1.44)	

Abbreviations: CI, confidence interval; HR, high risk; LR, low risk; OR, odds ratio.

^a^ Data are from 160 cases and 455 controls with available data and were adjusted for variables that influenced the effect estimate of the association between any HPV at s1 and HIV (ie, age group, alcohol consumption at enrollment, and transactional sex in the 3 months prior to first detection of HIV [time-updated variable]).

^b^ Data are from 158 cases and 454 controls with available data and were adjusted for the same variables as for adjusted analysis 1, as well as for the time-updated sexually transmitted infection variables *Chlamydia trachomatis* detection, *Neisseria gonorrhea* detection, and herpes simplex virus type 2 (HSV-2) detection. Women were classified as positive for *C. trachomatis*, *N. gonorrhea*, or HSV-2 if they had results for tests performed at the s0 and/or s − 1 visits and if the result from at least 1 visit was positive.

**Table 4.** Associations of Human Papillomavirus (HPV) Persistence, Clearance, and Acquisition With Subsequent Human Immunodeficiency Virus (HIV) Infection Among Women Who Had Valid HPV Test Results From at Least 2 Time Points

HPV Persistence, Clearance, Acquisition Prior to HIV Seroconversion^a^	Cases, No. (%) (n = 119)	Controls, No. (%) (n = 323)	Age-Adjusted Analysis		Adjusted Analysis 1^b^		Adjusted Analysis 2^c^	
			OR (95% CI)	*P* Value	OR (95% CI)	*P* Value	OR (95% CI)	*P* Value
Among all women with >1 valid result (at s0, s − 1, or s − 2)								
Uninfected	37 (31.1)	118 (36.5)	1	.771	1	0.765	1	.685
Any HPV clearance	53 (44.5)	134 (41.5)	1.17 (.68–2.00)		1.08 (.61–1.89)		0.85 (.46–1.55)	
HPV persistence for 6 mo	10 (8.4)	33 (10.2)	0.97 (.41–2.28)		0.94 (.39–2.27)		0.93 (.37–2.29)	
HPV acquisition only	19 (16.0)	38 (11.8)	1.42 (.68–2.95)		1.44 (.68–3.04)		1.33 (.61–2.90)	

Abbreviations: CI, confidence interval; OR, odds ratio.

^a^ Women who remained HPV negative at all available time points were classified as uninfected. Women with evidence of any HPV clearance, defined as a genotype-specific positive test result followed by a genotype-specific negative result, regardless of concurrent HPV persistence or acquisition, were classified as having any HPV clearance. Women with at least 2 genotype-specific positive test results at least 6 mo apart, with or without evidence of acquisition of other genotypes, were classified as having HPV persistence for 6 mo. Women who only had a genotype-specific negative test result followed by a positive result for the same genotype at a later time point, with no evidence of persistence or clearance of other genotypes, were classified as having HPV acquisition only.

^b^ Data are for 118 cases and 319 controls with available data and are adjusted for age group, alcohol consumption at enrollment as a linear variable, and transactional sex in the 3 months before the visit when HIV was first detected (ie, the same model as used for the analysis of the effect of prevalent HPV in Table 3; all other covariates were checked, but no other residual confounding was found).

^c^ Data are for 117 cases and 319 controls with available data and are adjusted for age, alcohol consumption at enrollment as a linear variable, transactional sex, and *Chlamydia trachomatis* and herpes simplex virus type 2 detection at time of or the visit before the first visit when HIV was detected. *Neisseria gonorrhea* detection was removed from the model to reduce the number of parameters, as it did not affect the adjusted OR.

Additional analyses of HR-HPV and LR-HPV types, analyses of nonavalent vaccine types (Table 3), and analyses restricted to the 87% of participants with s − 1 samples collected ≤6 months before HIV seroconversion (Supplementary Table 2) did not show notably different results.

### HPV Clearance or Persistence and HIV Acquisition

A total of 119 cases and 323 controls had data from at least 2 time points (groups B–D in Figure 1) and were included in the analysis of the association of HPV clearance and/or persistence with HIV acquisition. Among these women, 42% had evidence of clearance of an HPV genotype irrespective of the status of other genotype-specific infections (the “any clearance” group), 10% had evidence of 6-month persistence with an HPV genotype only (with no evidence of clearance, with/without acquisition of a different genotype), and 13% had evidence of acquisition of an HPV genotype only (with no concurrent clearance or persistence; Table 4). Among the 237 women with HPV data from an s − 2 visit, 94% had a s − 2 cervical sample collected at or within 12 months prior to the s0 visit.

Adjusted analyses based on data from all available time points did not show evidence of an association between the clearance, persistence, or acquisition of HPV infection and HIV acquisition (Table 4). Additional analyses restricted to s − 2 and s − 1 time points and excluding data from the s0 visit, when HIV infection could have influenced HPV infection, yielded somewhat larger effect estimates for all associations between HPV and HIV. However, CIs were wide, and none of the results were statistically significant (Supplementary Table 3).

## DISCUSSION

In these cohorts of Tanzanian and Ugandan women, there was no evidence of an association between HPV infection, clearance, persistence, or acquisition and the odds of HIV acquisition. These results stand in contrast to other observational studies in high-risk women, which found a 2-fold increased risk of HIV acquisition in those who were HPV positive [23]. There is a plausible mechanism by which HPV infection could increase the risk of HIV acquisition through mechanical or immunological changes in the cervix [20–22]. There is strong evidence of cofactor effects of other STIs on HIV transmission [39–46]. We sought to confirm the findings of previous studies and add to limited data on HPV clearance and subsequent HIV acquisition, but we were unable to provide evidence in our study populations for such association.

Strengths of our study include its size; with 161 cases and 464 controls, our study is the largest to date. In previous studies, the number of HIV seroconverters ranged from 4 to 145 women [24–26, 28, 47]. The original cohort studies used for our investigation had high retention rates, and the characteristics of cases and controls were very similar. Cases and controls were selected from the same populations and matched to account for different study sites and designs and for individuals' adherence to study procedures. A sensitivity analysis showed that results did not differ between studies using cervical swab specimens and the one study using CVL specimens to diagnose HPV infection. The risk of misclassification of HIV infection status was low, as all studies used confirmatory HIV tests.

There are a number of possible reasons why our results differ from those reported in the literature. Apart from the Ugandan women, who included self-identified sex workers, it could be considered that the remainder of the participants had a lower risk of HIV acquisition than women recruited in previous studies, some of which only recruited sex workers [24, 26]. However, >80% of both cases and controls in our study were HSV-2 seropositive. Although cases in previous studies had a similarly high HSV-2 prevalence, the prevalence in controls was significantly lower in previous studies [27, 47]. Interactions between HPV, other sexually transmitted pathogens, and the cervical-vaginal microbiome are unclear; however, the effect of STIs on HIV acquisition may diminish the relative effect of HPV. External quality control results from the ICO laboratory indicated fair agreement in the detection of HPV genotypes; nondifferential misclassification of HPV infection may have diluted the measured effect of HPV on HIV.

Across all exposure variables investigated, there was little evidence of confounding. No information on the relative risk status of the participants' sex partners was available and missing data precluded controlling for *T. vaginalis* infection, mobility, vaginal cleansing practices, and the presence of genital warts. However, when analyzing the available data on these variables, there were no significant associations with HIV status. Cases and controls were well balanced with regard to baseline STIs; however, residual confounding related to differences in sexual behavior cannot be excluded. The numbers of participants with clearance and 6-month persistence were small, and CIs were wide; the existence of a moderate effect of HPV on HIV acquisition cannot be ruled out.

Our study used samples that had been stored in freezers for 3–9 years. Samples that have been embedded in paraffin show good reproducibility [48, 49]; however, the durability of DNA in frozen swab aliquots or CVL specimens after long-term storage is undocumented. There is some evidence that detection of β-globin decreases over time, leading to a greater number of faint/invalid results and, therefore, potential misclassification of HPV infection status. The inclusion of s0 samples in our analysis could limit the temporal relevance of the analysis of clearance and persistence. Acquisition of HIV between the s − 1 and s0 time points may have increased the probability of HPV persistence and/or new acquisition of HPV, which was then detectable at the s0 time point [17]. However, these time points have been used in the 2 previous studies analyzing the effect of HPV clearance and persistence on HIV, with a similar prevalence of clearance (around 40%) [27, 28]. When restricting our analysis to the s − 2 and s − 1 time points, the effect estimates were larger; however, CIs were wide, and *P* values preclude any clear conclusions. All studies of the association between HPV and HIV infection potentially suffer from inadequate methods of defining HPV infection status. More research is needed to find biological markers of newly acquired infection, latent infection, clearance, and persistence.

In conclusion, the HIV epidemic remains a serious problem in sub-Saharan Africa, which simultaneously harbors the highest burden of HPV infection in the world [4]. If previous reports are accurate and HPV infection leads to a 2-fold increase in risk of HIV acquisition, the highly effective HPV vaccines could substantially influence rates of HIV acquisition in areas of high HPV incidence. Whereas previous studies suggested that HPV infection enhances the risk of HIV acquisition, our study, based on a large data set from 5 cohorts in East Africa, could not confirm this association; more research is needed to clarify these contrasting results. Given the contradictory evidence of an association between HPV and HIV, a cluster randomized controlled trial of phased delivery of HPV vaccine with monitored HIV infection incidence may be the only way of concluding whether there is a causal relationship between HPV infection and HIV acquisition.

## Supplementary Material

Supplementary materials are available at http://jid.oxfordjournals.org. Consisting of data provided by the author to benefit the reader, the posted materials are not copyedited and are the sole responsibility of the author, so questions or comments should be addressed to the author.

Supplementary Data

## References

[1] de Villiers EM Cross-roads in the classification of papillomaviruses. Virology 2013; 445:2–10. 23683837 10.1016/j.virol.2013.04.023

[2] International Agency for Research on Cancer (IARC). Human papillomaviruses. In: Biological agents. Vol 100 B: a review of human carcinogens. IARC monographs on the evaluation of carcinogenic risks to humans Lyon, France: IARC, 2012:255–313.

[3] Bosch FX, Broker TR, Forman D et al Comprehensive control of human papillomavirus infections and related diseases. Vaccine 2013; 31(Suppl 5):F1–31. 24331745 10.1016/j.vaccine.2013.10.001

[4] International Agency for Research on Cancer. GLOBOCAN 2012 [database online]. Cervical cancer incidence and mortality worldwide in 2012: summary. http://globocan.iarc.fr/Pages/fact_sheets_cancer.aspx Accessed 24 July 2014.

[5] Giuliano AR, Nyitray AG, Kreimer AR et al EUROGIN 2014 roadmap: Differences in HPV infection natural history, transmission, and HPV-related cancer incidence by gender and anatomic site of infection. Int J Cancer 2014; 136:2752–60. 25043222 10.1002/ijc.29082 PMC4297584

[6] Merck & Co. Merck Products: Vaccines. http://www.merck.com/product/vaccines/home.html. Accessed 5 March 2016.

[7] Chesson HW, Dunne EF, Hariri S, Markowitz LE The estimated lifetime probability of acquiring human papillomavirus in the United States. Sex Transm Dis 2014; 41:660–4. 25299412 10.1097/OLQ.0000000000000193 PMC6745688

[8] Ho GY, Burk RD, Klein S et al Persistent genital human papillomavirus infection as a risk factor for persistent cervical dysplasia. J Natl Cancer Inst 1995; 87:1365–71. 7658497 10.1093/jnci/87.18.1365

[9] Wallin KL, Wiklund F, Angstrom T et al Type-specific persistence of human papillomavirus DNA before the development of invasive cervical cancer. N Engl J Med 1999; 341:1633–8. 10572150 10.1056/NEJM199911253412201

[10] Winer RL, Hughes JP, Feng Q et al Early natural history of incident, type-specific human papillomavirus infections in newly sexually active young women. Cancer Epidemiol Biomarkers Prev 2011; 20:699–707. 21173170 10.1158/1055-9965.EPI-10-1108 PMC3078690

[11] Bruni L, Barrionuevo-Rosas L, Serrano B et al Human papillomavirus and related diseases in Tanzania. Summary Report [Accessed 17 March 2014]. Barcelona, Spain: Institut Catalonia d'Oncologia (ICO) Information Centre on HPV and Cancer (HPV Information Centre), 2014.

[12] Banura C, Mirembe FM, Katahoire AR, Namujju PB, Mbonye AK, Wabwire FM Epidemiology of HPV genotypes in Uganda and the role of the current preventive vaccines: A systematic review. Infect Agent Cancer 2011; 6:11. 21749691 10.1186/1750-9378-6-11 PMC3163594

[13] Korir A, Okerosi N, Ronoh V, Mutuma G, Parkin M Incidence of cancer in Nairobi, Kenya (2004–2008). Int J Cancer 2015; 137:2053–9. 26139540 10.1002/ijc.29674

[14] Watson-Jones D, Baisley K, Brown J et al High prevalence and incidence of human papillomavirus in a cohort of healthy young African female subjects. Sex Transm Infect 2013; 89:358–65. 23486859 10.1136/sextrans-2012-050685 PMC3717757

[15] Banura C, Franceschi S, Doorn LJ et al Infection with human papillomavirus and HIV among young women in Kampala, Uganda. J Infect Dis 2008; 197:555–62. 18237268 10.1086/526792

[16] Mbulawa ZZ, Marais DJ, Johnson LF, Coetzee D, Williamson AL Impact of human immunodeficiency virus on the natural history of human papillomavirus genital infection in South African men and women. J Infect Dis 2012; 206:15–27. 22517913 10.1093/infdis/jis299

[17] Wang C, Wright TC, Denny L, Kuhn L Rapid rise in detection of human papillomavirus (HPV) infection soon after incident HIV infection among South African women. J Infect Dis 2011; 203:479–86. 21216869 10.1093/infdis/jiq083 PMC3071227

[18] Massad LS, Evans CT, Minkoff H et al Natural history of grade 1 cervical intraepithelial neoplasia in women with human immunodeficiency virus. Obstet Gynecol 2004; 104:1077–85. 15516404 10.1097/01.AOG.0000143256.63961.c0

[19] Aubin F, Martin M, Puzenat E et al Genital human Papillomavirus infection in patients with autoimmune inflammatory diseases. Joint Bone Spine 2011; 78:460–5. 21570889 10.1016/j.jbspin.2011.03.002

[20] Leong CM, Doorbar J, Nindl I, Yoon HS, Hibma MH Deregulation of E-cadherin by human papillomavirus is not confined to high-risk, cancer-causing types. Br J Dermatol 2010; 163:1253–63. 20698848 10.1111/j.1365-2133.2010.09968.x

[21] Herfs M, Hubert P, Moutschen M, Delvenne P Mucosal junctions: open doors to HPV and HIV infections? Trends Microbiol 2011; 19:114–20. 21216598 10.1016/j.tim.2010.12.006

[22] Nicol AF, Fernandes AT, Grinsztejn B et al Distribution of immune cell subsets and cytokine-producing cells in the uterine cervix of human papillomavirus (HPV)-infected women: influence of HIV-1 coinfection. Diagn Mol Pathol 2005; 14:39–47. 15714063 10.1097/01.pas.0000143309.81183.6c

[23] Houlihan CF, Larke NL, Watson-Jones D et al Human papillomavirus infection and increased risk of HIV acquisition. A systematic review and meta-analysis. AIDS 2012; 26:2211–22. 22874522 10.1097/QAD.0b013e328358d908 PMC3831022

[24] Auvert B, Marais D, Lissouba P, Zarca K, Ramjee G, Williamson AL High-risk human papillomavirus is associated with HIV acquisition among South African female sex workers. Infect Dis Obstet Gynecol 2011; 2011:692012. 21804752 10.1155/2011/692012 PMC3143430

[25] Myer L, Denny L, Wright TC, Kuhn L Prospective study of hormonal contraception and women's risk of HIV infection in South Africa. Int J Epidemiol 2007; 36:166–74. 17175547 10.1093/ije/dyl251

[26] Veldhuijzen NJ, Vyankandondera J, van de Wijgert JH HIV acquisition is associated with prior high-risk human papillomavirus infection among high-risk women in Rwanda. AIDS 2010; 24:2289–92. 20613457 10.1097/QAD.0b013e32833cbb71

[27] Averbach SH, Gravitt PE, Nowak RG et al The association between cervical human papillomavirus infection and HIV acquisition among women in Zimbabwe. AIDS 2010; 24:1035–42. 20397287 10.1097/qad.0b013e3283377973 PMC3831602

[28] Smith-McCune KK, Shiboski S, Chirenje MZ et al Type-specific cervico-vaginal human papillomavirus infection increases risk of HIV acquisition independent of other sexually transmitted infections. PLoS One 2010; 5:e10094. 20386706 10.1371/journal.pone.0010094 PMC2851652

[29] Brown B, Davtyan M, Galea J, Chow E, Leon S, Klausner JD The role of human papillomavirus in human immunodeficiency virus acquisition in men who have sex with men: a review of the literature. Viruses 2012; 4:3851–8. 23250451 10.3390/v4123851 PMC3528294

[30] Tanser F, Jones KG, Viljoen J, Imrie J, Grapsa E, Newell ML Human papillomavirus seropositivity and subsequent risk of HIV acquisition in rural South African women. Sex Transm Dis 2013; 40:601–6. 23965780 10.1097/OLQ.0b013e3182918578 PMC4239474

[31] Abdool Karim Q, Libenberg L, Leask K et al HPV infection enhanced HIV acquisition in CAPRISA 004 trial participants in KwaZulu Natal, South Africa [abstract HPV15-0372]. Presented at: 30th International Papillomavirus Conference, Lisbon, Portugal, 17-21 September 2015.

[32] Rositch AF, Mao L, Hudgens MG et al Risk of HIV acquisition among circumcised and uncircumcised young men with penile human papillomavirus infection. AIDS 2014; 28:745–52. 24149088 10.1097/QAD.0000000000000092 PMC4074250

[33] Tobian AA, Grabowski MK, Kigozi G et al Human papillomavirus clearance among males is associated with HIV acquisition and increased dendritic cell density in the foreskin. J Infect Dis 2013; 207:1713–22. 23345339 10.1093/infdis/jit035 PMC3636782

[34] Watson-Jones D, Weiss HA, Rusizoka M et al Effect of herpes simplex suppression on incidence of HIV among women in Tanzania. N Engl J Med 2008; 358:1560–71. 18337596 10.1056/NEJMoa0800260 PMC2643126

[35] Vandepitte J, Bukenya J, Weiss HA et al HIV and other sexually transmitted infections in a cohort of women involved in high-risk sexual behavior in Kampala, Uganda. Sex Transm Dis 2011; 38:316–23. 23330152 PMC3920055

[36] Vallely A, Hambleton IR, Kasindi S et al Are women who work in bars, guesthouses and similar facilities a suitable study population for vaginal microbicide trials in Africa? PLoS One 2010; 5:e10661. 20498833 10.1371/journal.pone.0010661 PMC2871045

[37] Kapiga SH, Ewings FM, Ao T et al The epidemiology of HIV and HSV-2 infections among women participating in microbicide and vaccine feasibility studies in Northern Tanzania. PLoS One 2013; 8:e68825. 23874780 10.1371/journal.pone.0068825 PMC3715536

[38] Roche Molecular Diagnostics. Linear Array HPV genotyping test. http://molecular.roche.com/assays/Pages/LINEARARRAYHPVGenotypingTest.aspx Accessed 6 August 2014.

[39] Baeten JM, Benki S, Chohan V et al Hormonal contraceptive use, herpes simplex virus infection, and risk of HIV-1 acquisition among Kenyan women. Aids 2007; 21:1771–7. 17690576 10.1097/QAD.0b013e328270388a

[40] Wald A, Link K Risk of human immunodeficiency virus infection in herpes simplex virus type 2-seropositive persons: a meta-analysis. J Infect Dis 2002; 185:45–52. 11756980 10.1086/338231

[41] del Mar Pujades Rodriguez M, Obasi A, Mosha F et al Herpes simplex virus type 2 infection increases HIV incidence: a prospective study in rural Tanzania. AIDS 2002; 16:451–62. 11834958 10.1097/00002030-200202150-00018

[42] Van Der Pol B, Kwok C, Pierre-Louis B et al Trichomonas vaginalis infection and human immunodeficiency virus acquisition in African women. J Infect Dis 2008; 197:548–54. 18275275 10.1086/526496

[43] Laga M, Manoka A, Kivuvu M et al Non-ulcerative sexually transmitted diseases as risk factors for HIV-1 transmission in women: results from a cohort study. AIDS 1993; 7:95–102. 8442924 10.1097/00002030-199301000-00015

[44] Kapiga SH, Sam NE, Bang H et al The role of herpes simplex virus type 2 and other genital infections in the acquisition of HIV-1 among high-risk women in northern Tanzania. J Infect Dis 2007; 195:1260–9. 17396994 10.1086/513566

[45] van de Wijgert JH, Morrison CS, Brown J et al Disentangling contributions of reproductive tract infections to HIV acquisition in African Women. Sex Transm Dis 2009; 36:357–64. 19434010 10.1097/OLQ.0b013e3181a4f695

[46] NgBE, ButlerLM, HorvathT, RutherfordGW Population-based biomedical sexually transmitted infection control interventions for reducing HIV infection. Cochrane Database Syst Rev 2011; doi:10.1002/14651858.CD001220.pub3.10.1002/14651858.CD001220.pub321412869

[47] Low AJ, Clayton T, Konate I et al Genital warts and infection with human immunodeficiency virus in high-risk women in Burkina Faso: a longitudinal study. BMC Infect Dis 2011; 11:20. 21251265 10.1186/1471-2334-11-20 PMC3031229

[48] Odida M, de Sanjose S, Sandin S et al Comparison of human papillomavirus detection between freshly frozen tissue and paraffin embedded tissue of invasive cervical cancer. Infect Agent Cancer 2010; 5:15. 20846370 10.1186/1750-9378-5-15 PMC2954863

[49] Siriaunkgul S, Suwiwat S, Settakorn J et al HPV genotyping in cervical cancer in Northern Thailand: adapting the linear array HPV assay for use on paraffin-embedded tissue. Gynecol Oncol 2008; 108:555–60. 18199473 10.1016/j.ygyno.2007.11.016

