# Supplementary material for: The Association Between Cervical Human Papillomavirus Infection and Subsequent HIV Acquisition in Tanzanian and Ugandan Women: A Nested Case-Control Study
Source: J Infect Dis. 2016 Mar 6;214(1):87–95. doi: 10.1093/infdis/jiw094 (PMC4907415; doi:10.1093/infdis/jiw094)
Supplement: Supplementary Data [file supp_214_1_87__index.html]

The association between cervical human papillomavirus infection and subsequent HIV acquisition in Tanzanian and Ugandan women: a nested case-control study — The Association Between Cervical Human Papillomavirus Infection and Subsequent HIV Acquisition in Tanzanian and Ugandan Women: A Nested Case-Control Study — The Association Between Cervical Human Papillomavirus Infection and Subsequent HIV Acquisition in Tanzanian and Ugandan Women: A Nested Case-Control Study — Supplementary Data 

# The Association Between Cervical Human Papillomavirus Infection and Subsequent HIV Acquisition in Tanzanian and Ugandan Women: A Nested Case-Control Study

## Supplementary Data

Supplementary Data

- Supplementary Table 1 - docx file
- Supplementary Table 2 - docx file
- Supplementary Table 3 - docx file
