# Supplementary material for: The Association Between Cervical Human Papillomavirus Infection and Subsequent HIV Acquisition in Tanzanian and Ugandan Women: A Nested Case-Control Study
Source: J Infect Dis. 2016 Mar 6;214(1):87–95. doi: 10.1093/infdis/jiw094 (PMC4907415; doi:10.1093/infdis/jiw094)
Supplement: Supplementary Data [file supp_jiw094_jiw094supp_table1.docx]

**Supplementary Table 1: External quality assurance results; 102 samples tested at the National Institute for Medical Research (NIMR), Mwanza were re-tested by the Catalan Institute of Oncology (ICO), Barcelona^a^.**

| **HPV genotypes** | **ICO** | |  |
| --- | --- | --- | --- |
| **NIMR** | **-** | **+** | **Total** |
| **-** | 2869 | 51 | 2920 |
| **+** | 41 | 108 | 149 |
| **Total** | 2910 | 159 | 3069 |

^a^ 102 samples were randomly selected for re-testing at the ICO laboratory using Roche Linear Array for 37 different genotypes; 9 samples gave inhibited results on retesting at ICO; 4 genotypes were excluded from comparison due to differences in reporting practices at the 2 institutions (HPV 52, 33, 35, 58). This gave 3069 pairs of results for quality assurance (93 samples x 33 genotypes). Overall percentage agreement: 97%; Positive percentage agreement: 68%; Negative percentage agreement: 99%; Kappa statistic for agreement between the laboratories: 0.69.
