# Supplementary material for: The Association Between Cervical Human Papillomavirus Infection and Subsequent HIV Acquisition in Tanzanian and Ugandan Women: A Nested Case-Control Study
Source: J Infect Dis. 2016 Mar 6;214(1):87–95. doi: 10.1093/infdis/jiw094 (PMC4907415; doi:10.1093/infdis/jiw094)
Supplement: Supplementary Data [file supp_jiw094_jiw094supp_table2.docx]

**Supplementary table 2:** results when restricted to s-1 samples collected within 6 months of first detection of HIV.

| **HPV infection status at the study visit preceding HIV seroconversion (s-1) among samples within 6 months** | **Cases**  **(%)**  **N=140** | **Controls (%)**  **N=405** | | **Age-adjusted OR (95%CI)**  **(p-value)** | **aOR1^a^**  **(95%CI)**  **(N=140/398)**  **(p-value)** | **aOR2^b^ (95%CI)**  **(N=138/397)**  **(p-value)** |
| --- | --- | --- | --- | --- | --- | --- |
| **Any HPV** |  | |  | 0.540 | 0.590 | 0.599 |
| HPV uninfected | 67 (47.9) | 211 (52.1) | | 1 | 1 | 1 |
| **Any HPV** infection | 73 (52.1) | 194 (47.9) | | 1.14  (0.75-1.73) | 1.13  (0.73-1.75) | 1.13  (0.71-1.82) |
| **Nonavalent vaccine types** |  |  | | 0.680 | 0.612 | 0.625 |
| HPV uninfected | 67 (47.9) | 211 (52.1) | | 1 | 1 | 1 |
| **Nonavalent** HPV infection | 35 (25.0) | 94 (23.2) | | 1.04  (0.63-1.73) | 1.00  (0.59-1.69) | 1.00  (0.57-1.75) |
| **Other HPV+ infection** | 38 (27.1) | 100 (24.7) | | 1.24  (0.76-2.04) | 1.27  (0.76-2.14) | 1.28  (0.74-2.22) |
| **HR/LR HPV infection** |  |  | | 0.669 | 0.738 | 0.658 |
| HPV uninfected | 67 (47.9) | 211 (52.1) | | 1 | 1 | 1 |
| HR HPV infection only | 23 (16.4) | 61 (15.1) | | 1.09  (0.61-1.96) | 1.11  (0.60-2.03) | 1.20  (0.63-2.27) |
| LR HPV infection only | 30 (21.4) | 71 (17.5) | | 1.42  (0.81-2.49) | 1.45  (0.80-2.62) | 1.46  (0.78-2.73) |
| HR-LR HPV co-infection | 20 (14.3) | 62 (15.3) | | 0.94  (0.51-1.70) | 0.89  (0.47-1.66) | 0.83  (0.43-1.60) |

^a^ aOR1 is adjusted for variables which influenced the effect estimate of the association between any HPV at s1 and HIV i.e. age group, alcohol consumption at enrolment, and transactional sex in the 3 months prior to first detection of HIV (time updated variable).

^b^ aOR2 is adjusted for those variables in aOR1 and additionally the time-updated STI variables CT, NG, HSV-2. Women were classified as positive for CT, NG, or HSV2 if they had results for at least one of the s0 or the s-1 visits and at least one result was positive.
