# Supplementary material for: The Association Between Cervical Human Papillomavirus Infection and Subsequent HIV Acquisition in Tanzanian and Ugandan Women: A Nested Case-Control Study
Source: J Infect Dis. 2016 Mar 6;214(1):87–95. doi: 10.1093/infdis/jiw094 (PMC4907415; doi:10.1093/infdis/jiw094)
Supplement: Supplementary Data [file supp_jiw094_jiw094supp_table3.docx]

**Supplementary Table 3: Associations of HPV persistence, clearance and acquisition with subsequent HIV infection among women who had valid HPV results for at least 2 time points, restricted to selected time points.**

| **HPV persistence, clearance, acquisition prior to HIV seroconversion^a^** | **Cases**  **(%)** | **Controls (%)** | **Age-adjusted OR (95%CI)**  **(p-value)** | **aOR1^b^**  **(95%CI)**  **(p-value)** | **aOR2^c^**  **(95%CI)**  **(p-value)** |
| --- | --- | --- | --- | --- | --- |
| Among s-1 and s0 samples only | N=78 | N=209 | 0.664 | N=77/209  0.482 | N=77/209  0.226 |
| Uninfected | 32  (41.0) | 84  (40.2) | 1 | 1 | 1 |
| Any HPV clearance | 26 (33.3) | 79  (37.8) | 0.79  (0.42-1.49) | 0.70  (0.36-1.36) | 0.54  (0.26-1.10) |
| HPV 6m persistence only | 4  (5.1) | 15  (7.2) | 0.60  (0.17-2.08) | 0.52  (0.14-1.95) | 0.46  (0.12-1.74) |
| HPV Acquisition only | 16  (20.5) | 31  (14.8) | 1.17  (0.53-2.60) | 1.16  (0.51-2.63) | 1.08  (0.45-2.56) |
| Among s-2 and s-1 samples only | N=62 | N=169 | 0.547 | N=62/165  0.612 | N=61/165  0.828 |
| Uninfected | 17  (27.4) | 67  (39.6) | 1 | 1 | 1 |
| Any HPV clearance | 31  (50.0) | 67  (39.6) | 1.83  (0.80-4.23) | 1.70  (0.71-4.10) | 1.08  (0.41-2.85) |
| HPV 6m persistence only | 5  (8.1) | 14  (8.3) | 1.68  (0.46-6.20) | 2.09  (0.54-8.04) | 1.93  (0.46-8.02) |
| HPV Acquisition only | 9  (14.5) | 21  (12.4) | 1.51  (0.52-4.35) | 1.66  (0.56-4.97) | 1.17  (0.35-3.87) |

^a^ HPV classifications: ‘Uninfected’ consists of women who remained HPV negative at all available time points; ‘Any HPV clearance’ includes women who had evidence of any HPV clearance, a genotype specific HPV positive result followed by a genotype specific HPV negative result, regardless of concurrent HPV persistence or acquisition; ‘HPV 6m persistence’ category included women with at least 2 genotype specific positive results at least 6 months apart with or without evidence of acquisition of other genotypes; ‘Acquisition only’ included women who only had evidence of a genotype specific negative HPV result followed by a positive result for the same genotype at a later time point with no evidence of persistence or clearance of other genotypes.

^b^ aOR1 adjusted for age group, alcohol consumption at enrolment as a linear variable and transactional sex in the 3 months prior to first detection of HIV (i.e. the same model as used for the analysis of the effect of prevalent HPV in Table 3; all other covariates were checked but no other residual confounding was found).

^c^ aOR2 is adjusted for age, alcohol consumption at enrolment as a linear variable, transactional sex, CT and HSV2 detected at first detection of HIV or the previous visit. NG was removed from the model to reduce the number of parameters as it did not affect the aOR.
